# Human endogenous retroviruses in schizophrenia: clinical evidence, molecular mechanisms, and implications

**DOI:** 10.3389/fcimb.2025.1677212

**Published:** 2025-10-22

**Authors:** Mengyu Zhang, Xiaoge Wang, Yun Liu, Chenxuan Bao, Qing Gao, Lingxiang Mao

**Affiliations:** Department of Laboratory Medicine, Affiliated Kunshan Hospital of Jiangsu University, Kunshan, Jiangsu, China

**Keywords:** human endogenous retroviruses, schizophrenia, neuroinflammation, gene-environment interactions, synaptic dysfunction, precision psychiatry

## Abstract

Human endogenous retroviruses (HERVs), comprising 8% of the human genome, are implicated in schizophrenia, a complex psychiatric disorder driven by genetic, epigenetic, and environmental factors. This review examines the role of HERVs in schizophrenia pathogenesis. We synthesized clinical evidence, molecular mechanisms, and gene-environment interactions from studies on HERVs expression in schizophrenia, focusing on HERV-W and HERV-K in peripheral blood, cerebrospinal fluid, and brain tissues. Elevated HERV-W and HERV-K env and gag transcripts are consistently observed in individuals with schizophrenia, indicating potential diagnostic biomarkers. HERVs contribute to neuroinflammation, neurotoxicity, and epigenetic dysregulation of risk genes. The HERV-W env activates the Toll-like receptor 4 (TLR4)/MyD88 pathway, disrupting glutamatergic and dopaminergic signaling, leading to synaptic dysfunction and neuronal apoptosis. Environmental triggers, such as viral infections and early-life stress, activate HERVs, linking genetic and environmental risks. Variability in HERV expression across disease stages highlights the need for standardized assays and longitudinal studies. Emerging technologies and preclinical models targeting HERV-W env offer promise for developing novel diagnostics and therapies. HERVs serve as pivotal mediators of schizophrenia’s etiology, advancing precision psychiatry through biomarker and therapeutic innovation.

## 1 Introduction

Approximately 8% of the human genome comprises sequences derived from retroviral integrations, collectively termed HERVs (Kury et al., 2018). Retroviruses, characterized by their RNA genomes, integrate into host chromosomes via reverse transcriptase. These exist in exogenous or endogenous forms, or both. Exogenous retroviruses infect cells through specific receptors, whereas endogenous retroviruses are embedded in the genomes of all host cells and inherited in a Mendelian fashion. Owing to mutations, frameshifts, and deletions, most HERVs have lost their coding potential.

Schizophrenia is a severe psychiatric disorder characterized by positive symptoms (e.g., hallucinations, delusions, disordered thinking and behavior) and negative symptoms (e.g., apathy, anhedonia, social withdrawal) (McCutcheon et al., 2020). Its etiology remains incompletely understood, with contributions from genetic and environmental factors (Sullivan et al., 2003). Genetic studies have identified 287 genomic loci associated with schizophrenia risk, primarily affecting neuronal gene expression, synaptic function, neurotransmitter signaling, neurodevelopment, immune responses, and epigenetic regulation (Chang et al., 2017; Sekar et al., 2016; Trubetskoy et al., 2022). Aberrant gene regulation during fetal brain development may influence postnatal brain phenotypes, with open chromatin regions enriched for schizophrenia risk alleles (Hill and B, 2012; Tao et al., 2014). Environmental risk factors, including socioeconomic stressors (e.g., poverty, inequality, urban density) (Kirkbride et al., 2014); the season of birth (Szoke et al., 2024); maternal infections during pregnancy (e.g., influenza, rubella, herpes simplex virus, cytomegalovirus, hepatitis B, and *Toxoplasma gondii*) (Illescas-Montes et al., 2019; Liu et al., 2017; Mohebalizadeh et al., 2024); and immune dysregulation, also contribute to schizophrenia risk.

HERVs occupy a unique position at the intersection of genetic, epigenetic, and environmental risk factors, offering a valuable perspective for exploring the complex etiology of schizophrenia. As genomic regulatory elements, HERVs can act as enhancers or promoters, influencing the expression of genes critical for neurodevelopment and synaptic function, such as brain-derived neurotrophic factor (*BDNF*) and disrupted in schizophrenia 1 (*DISC1*) (Chen et al., 2018; Qin et al., 2016). Additionally, HERVs activation is often linked to viral infections, inflammation, and epigenetic dysregulation, all of which are pivotal in schizophrenia pathophysiology (Rangel et al., 2022; Wang et al., 2021). For instance, the HERV-W env protein can trigger neuroinflammation by activating microglia and proinflammatory cytokines, potentially exacerbating schizophrenia pathology (Wang et al., 2021). Numerous clinical studies have reported elevated HERV expression in the peripheral blood and brain tissues of individuals with schizophrenia, suggesting its potential as a biomarker (Rangel et al., 2024; Wu et al., 2023a, 2023; Yolken, 2004; Zhang et al., 2024). Thus, HERVs offer a molecular entry point for studying schizophrenia and hold promise as targets for novel diagnostic and therapeutic strategies. This review synthesizes clinical evidence, molecular mechanisms, and risk factor associations to elucidate the role of HERVs in schizophrenia and their significance in psychiatric research.

## 2 Biology of HERVs

### 2.1 Structure and classification of HERVs

**Figure 1:**
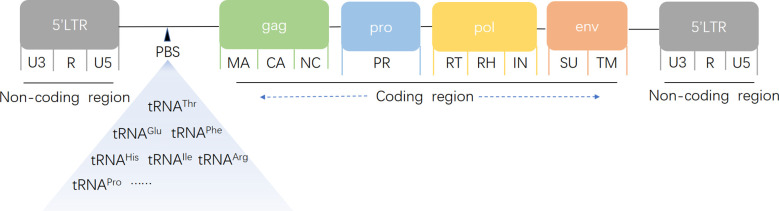
Schematic structure of a human endogenous retrovirus genome. gag encodes capsid (CA), nucleocapsid (NC), and matrix protein (MA); pro encodes protease (PR); pol encodes reverse transcriptase (RT), RNase H (RH), and integrase (IN); and env encodes surface (SU) and transmembrane (TM) units. A noncoding primer-binding site (PBS) specific to tRNA is located between the 5′ LTR and the first gag codon. LTRs consist of unique 3′ (U3), repeat (R), and unique 5′ (U5) regions.

HERVs are a subclass of transposable elements (TEs) that mobilize within the genome via an RNA intermediate through a “copy-and-paste” mechanism (Vargiu et al., 2016). Structurally, HERVs contain essential viral genes, such as gag (encoding capsid proteins), pro (encoding proteases), pol (encoding reverse transcriptase), and env (encoding envelope proteins), which are flanked by long terminal repeats (LTRs) (**Figure 1**). LTRs, noncoding regions with promoter and enhancer activity, serve as influential regulatory modules for both HERVs and adjacent host genes. However, owing to negative selection and mutation accumulation (e.g., deletions, stop codons, frameshifts), most HERVs are transcriptionally silent. Historically considered “junk DNA” (Ochoa Thomas et al., 2020), recent evidence suggests that HERVs can regulate gene expression under specific physiological conditions (Shin et al., 2013), impacting transcriptional activity (Fueyo et al., 2022; Lawson et al., 2023) and genomic stability through rearrangements or insertional mutagenesis (Hughes and Coffin, 2001).

On the basis of their sequence characteristics, HERVs are classified into three major groups: gamma-like, beta-like, and spuma-like retroviruses (Tristem, 2003). HERV nomenclature typically reflects the tRNA specificity of their primer-binding sites and LTR structure (Heidmann et al., 2018). For example, HERV-W uses a tryptophan-specific tRNA primer, HERV-H uses a histidine-specific tRNA, and HERV-K uses a lysine-specific tRNA (Dolei, 2006). **Table 1** summarizes the classification, tRNA primer specificity, and key features of the 26 HERV lineages.

**Table 1:** Classification and characteristics of HERV families.

HERV class	HERV lineages	tRNA primer specificity	Key structural features	Physiological role	Disease associations
Class I (gamma-like)	HERV.Z69907	Not Determined (ND)	Contains gag, pol, env, LTRs	Not well-defined, potentially involved in gene regulation	Not established^1^
	HERV.ADP	tRNA^Thr^(?)	Contains LTRs, may lack some coding genes	Possibly involved in embryonic development	
	HERV.E	tRNA^Glu^	Contains gag, pol, env, LTRs	Regulation of gene expression	Cancer
	HERV.F	tRNA^Phe^		May influence genomic stability	Not established
	HERV.F (type b)	tRNA^Phe^		Not established	
	HERV.FRD	tRNA^His^	Contains env, encodes Syncytin-2	Mediates trophoblast cell fusion in placenta	
	HERV.H	tRNA^His^	Highly expressed in embryonic stem cells	Regulates pluripotency in embryonic stem cells	Cancer, Schizophrenia
	HERV.H49C23	No LTRs	Atypical structure, limited functionality	Not established	Not established
	HERV.I	tRNA^Ile^	Contains gag, pol, env, LTRs	Possibly involved in immune regulation	
	RRHERV.I	tRNA^Ile^	Contains LTRs, may lack some coding genes	Not established	
	ERV-9	tRNA^Arg^	Contains gag, pol, env, LTRs	Potentially involved in gene regulation	Schizophrenia
	HERV.F (type c)	tRNA^Phe^		Not established	Not established
	HERV.P	tRNA^Pro^			
	HERV.R	tRNA^Arg^			
	HERV.R (type b)	tRNA^Arg^			
	HERV.T	tRNA^Thr^			
	HERV.W	tRNA^Trp^	Contains env (ERVW-1, Syncytin-1), LTRs with promoter/enhancer activity	Placentation, immune tolerance regulation	Schizophrenia, Multiple Sclerosis, Cancer
	HERV.XA	tRNA^Phe^	Contains gag, pol, env, LTRs	Not established	Not established
Class II (beta-like)	HERV.K.HML1-4	tRNA^Lys^	Relatively intact genome, capable of producing functional viral proteins	Potentially involved in innate immune responses	Schizophrenia, Cancer
	HERV.K.HMLS	tRNA^Ile^	Contains gag, pol, env, LTRs	Not established	Not established
	HERV.K.HML6	tRNA^Lys^			
	HERV.K.HML9	ND			
Class III (spuma-like)	HERV.L	tRNA^Leu^			
	HERV.S	tRNA^Ser^			
	HERV.U2	ND			
	HERV.U3	ND			

^1^Where physiological roles or disease associations are listed as 'Not established,' further research is needed to clarify their involvement.

### 2.2 Physiological functions and regulatory mechanisms of HERVs

HERVs are relics of ancient exogenous retroviral infections that have been integrated into the germline, with most having lost replication capacity due to mutations over millions of years. However, some retain functional sequences that contribute to essential physiological processes, particularly during embryogenesis and placentation (Lavialle et al., 2013). For instance, the HERV-W element on chromosome 7q21 encodes Syncytin-1 (ERVW-1), integrated approximately 12–80 million years ago, which facilitates trophoblast cell fusion during placental development (Mi et al., 2000) and regulates maternal immune tolerance via exosomal sorting (Tolosa et al., 2012). HERV-H elements, which are highly expressed in embryonic stem cells, regulate pluripotency (Santoni et al., 2012). HERV-K (HML-2 subtype) retains relatively intact genomic sequences, enabling the production of functional viral proteins or particles under specific conditions (Shin et al., 2023). HERVs also modulate innate immune responses, acting as endogenous sensors of viral infections by interacting with pattern recognition receptors (e.g., Toll-like receptors, RIG-I-like receptors), initiating antiviral pathways, and influencing cytokine production (Russ and Iordanskiy, 2023). Additionally, HERVs contribute to genetic diversity and evolution by providing regulatory sequences (e.g., promoters, enhancers, alternative splice sites) that modulate adjacent gene expression and drive genomic recombination (Jern and Coffin, 2008).

## 3 Aberrant activation of HERVs in disease

Aberrant HERVs reactivation threatens genomic integrity and contributes to the development of various diseases, including psychiatric disorders. Environmental stimuli, including viral infections (Canli, 2019), pharmacological agents (Liu et al., 2017) and epigenetic modifications (van der Kuyl, 2012), can activate HERVs, leading to DNA damage, inflammation, and neurodegeneration (Chuong et al., 2017; Ochoa Thomas et al., 2020). HERVs are implicated in multifactorial diseases characterized by immune dysregulation, such as cancer, inflammatory disorders, and neurological and psychiatric conditions (Grandi and Tramontano, 2018; Groger and Cynis, 2018; Kury et al., 2018; Matteucci et al., 2018). For example, HERV-K LTRs act as enhancers in breast, lung, and colorectal cancers, driving oncogene expression and tumorigenesis (Fan and Cui, 2023). In addition to acting as enhancers in cancer and driving oncogene expression and tumorigenesis, HERVs can also function directly as oncogenes. According to the classical definition, oncogenes are genes that, through mutation, overexpression, or aberrant activation, promote cell proliferation, inhibit apoptosis, or induce metastasis. Research has demonstrated that specific mutations in the 3’-long terminal repeat (LTR) region of the HERV-W family on chromosome 7 enhance binding to the transcription factor c-Myb, significantly upregulating syncytin-1 expression. In urothelial cell carcinoma of the bladder, syncytin-1 overexpression directly drives cancer cell proliferation, survival, and tumorigenesis (Yu et al., 2014). Similar to the mutational activation of classical oncogenes, HERV elements transition from “passive” sequences to core drivers of tumor development. Similarly, in hepatocellular carcinoma, syncytin-1 overexpression enhances cell proliferation, metastasis, invasiveness, and doxorubicin resistance via activation of the MEK/ERK signaling pathway (Zhou et al., 2021). HERVs also exacerbate infectious and chronic inflammatory diseases by stimulating proinflammatory cytokine production (e.g., interleukin-6 [IL-6] and tumor necrosis factor-alpha [TNF-α])) (Rangel et al., 2022). In neurodegenerative and neuroinflammatory disorders, HERV-W env is overexpressed in multiple sclerosis lesions, and this overexpression is correlated with microglial activation and neuroinflammation (Kury et al., 2018). HERVs also exhibit transcriptional activity in Alzheimer’s and Parkinson’s diseases (Adler et al., 2024).

The causality of HERVs activation in disease remains debated. The evidence supporting causality includes HERV-W env overexpression preceding multiple sclerosis symptoms (Kury et al., 2018) and HERV-K-driven oncogene activation in early tumorigenesis (Mao et al., 2021). Conversely, HERV activation may be a consequence of inflammation or infection, establishing a feedback loop that exacerbates disease progression (Rangel et al., 2022). The HervD Atlas database highlights bidirectional associations between HERVs and disease, where HERVs may exacerbate pathology or result from it (Li C. et al., 2024). Given their role in neuroinflammation and psychiatric disorders, HERVs are a compelling focus for schizophrenia research, a disorder characterized by gene-environment interactions, neurodevelopmental abnormalities, and synaptic dysfunction (Duarte et al., 2024). Elevated HERVs expression in individuals with schizophrenia’ blood and brain tissues suggests a role in mediating interactions among infection, immune dysregulation, and genetic risk (Tamouza et al., 2021). Thus, schizophrenia provides a valuable model for investigating the pathological mechanisms of HERVs in psychiatric disorders.

## 4 Clinical evidence of HERVs expression in schizophrenia

### 4.1 HERVs expression in peripheral and central nervous systems

Numerous clinical studies have demonstrated aberrant HERVs expression in the peripheral blood and central nervous system (CNS) of individuals with schizophrenia (**Table 2**). In peripheral blood, HERV-W env and gag transcripts and proteins are detectable in both healthy controls and first-episode schizophrenia (FES) patients, with significantly higher levels in patients (Perron et al., 2008; Rangel et al., 2024; Wu et al., 2023a, b; Yao et al., 2008; Zhang et al., 2024). One study reported HERV-W env homologous mRNA sequences in the plasma of 42 out of 118 recent-onset individuals with schizophrenia, but not in controls. This discrepancy may be due to differences in sample type (plasma vs. whole blood) and target sequence (cloned vs. canonical HERV-W env transcripts). Plasma-free HERV RNA may reflect recent viral activity, while cellular transcripts are more stable and detectable (Rangel et al., 2024). A reduced HERV-W env gene copy number (Perron et al., 2012) and detection of reverse transcriptase activity(W. Huang et al., 2011, 2006) further suggest enhanced HERV transcriptional and translational activity in individuals with schizophrenia.

**Table 2:** Clinical evidence of HERV expression in schizophrenia.

Study reference	Sample type	HERV type	Expression pattern	Patient cohort	Key findings	Limitations
Wu et al., 2023 (Wu et al., 2023a)	Peripheral Blood (Whole Blood, Plasma)	HERV-W	Significant upregulation of ERVWE1 transcripts	First-Episode Schizophrenia (FES)	ERVWE1 significantly upregulated in FES patients (15 whole blood, 44 plasma) vs. controls (14 whole blood, 37 plasma), positively correlated with HTR1B, *ALKBH5*, and *Arc*.	Small sample size (15 FES vs. 14 controls, whole blood; 44 FES vs. 37 controls, plasma). No demographic differences, but potential confounders not fully addressed.
Wu et al., 2023 (Wu et al., 2023b)	Peripheral Blood (Plasma)		Significant upregulation of HERV-W env protein	FES	HERV-W env protein significantly elevated in plasma of recent-onset schizophrenia patients (n=44) vs. controls (n=37) by ELISA, negatively correlated with reduced 5-HT4R levels.	Small sample size (44 FES vs. 37 controls). Potential confounders (e.g., medication) not fully addressed, although age, gender, body mass index (BMI) controlled.
Rangel et al., 2024 (Rangel et al., 2024)			Significant upregulation of HERV-W env transcripts	Schizophrenia (nonpsychotic phase)	HERV-W env transcripts significantly higher in schizophrenia patients (n=24) vs. controls (n=46) (p<0.01) by using quantitative reverse transcription polymerase chain reaction (qRT-PCR). Elevated TNF-α, IL-10 (p=0.01), reduced IFN-γ, IL-2 (p=0.05) in schizophrenia, but no correlation with HERV-W env.	Small sample size (24 schizophrenia vs. 46 controls). Medicated patients may confound results. Limited to nonpsychotic phase, not reflecting acute states.
Zhang et al., 2024 (Zhang et al., 2024)			Significant upregulation of ERVW-1 transcripts	Schizophrenia (Stage not specified)	ERVW-1 transcripts significantly elevated in schizophrenia plasma (n=44) vs. co ntrols (n=37) (p<0.05) by qRT-PCR, negatively correlated with reduced *GPX4* and *SLC3A2* (p<0.05), supporting ferroptosis role.	Small sample size (44 schizophrenia vs. 37 controls). Stage unclear, likely FES. Potential confounders (e.g., medication) not fully addressed, although age, gender controlled.
Perron et al., 2008 (Perron et al., 2008)	Peripheral Blood (Whole Blood)		Significant upregulation of env and gag transcripts	FES	HERV-W env and gag antigenemia higher in schizophrenia patients than healthy controls, suggesting potential as a biomarker	Small sample size, specificity requires further validation
Yao et al., 2008 (Yao et al., 2008)			Elevated transcript levels		HERV-W transcripts significantly higher in blood cells of FES patients compared to healthy controls	Did not explore differences across disease stages, limited to single sample type
Perron et al., 2012 (Perron et al., 2012)			Reduced env gene copy number, enhanced reverse transcriptase activity	Schizophrenia (Stage not specified)	Increased HERV-W transcriptional and translational activity, potentially linked to inflammation	Limited sample size, did not distinguish between FES and MES
Mak et al., 2019 (Mak et al., 2019)	Peripheral Blood	HERV-K	Reduced methylation levels	FES	HERV-K methylation significantly lower in FES patients compared to healthy controls, independent of antipsychotic drug dosage	Limited sample size, differences in MES patients not explored
Tamouza et al., 2021 (Tamouza et al., 2021)		HERV-W	ENV protein positivity	Schizophrenia (Stage not specified)	HERV-W ENV-positive patients exhibited more manic symptoms and higher chlorpromazine dosage	Disease stage not specified, causality requires further validation
Huang et al., 2006 (Huang et al., 2006)			Detection of pol RNA and protein		HERV pol RNA and protein detected in blood, suggesting reverse transcriptase activity	Patient clinical characteristics not specified, detection method specificity needs optimization
Otowa et al., 2006 (Otowa et al., 2006)		HERV-K115	No significant difference in HERV-K115 insertion frequency		HERV-K115 insertion frequency similar in schizophrenia (8.4%, n=119) vs. controls (9.4%, n=117) (p>0.05) by PCR; marginal link to younger onset in schizophrenia (p=0.057).	No HERV expression data, only insertion polymorphism. Small sample size (119 schizophrenia vs. 117 controls). Schizophrenia stage unspecified, mixing acute/chronic cases. Limited power for age-at-onset analysis.
Canuti et al., 2015 (Canuti et al., 2015a)	Peripheral Blood (Serum)	Multiple HERVs	No evidence of endogenous retrovirus involvement	FES(Predominantly negative symptoms)	Viral metagenomics found no HERV involvement, possibly due to low inflammation in patients with negative symptoms	Sample biased toward negative-symptom patients, small sample size
Karlsson et al., 2001 (Karlsson et al., 2001)	Cerebrospinal Fluid, Brain Tissue	HERV (Unspecified)	Detection of retroviral RNA	Schizophrenia (Stage not specified)	Retroviral RNA detected in cerebrospinal fluid and brain tissue, indicating central expression	HERV type not specified, limited sample size
Yolken et al., 2004 (Yolken, 2004)	Cerebrospinal Fluid	HERV (pol homologous sequences)	Pol homologous sequences detected in 28.6% of samples	FES	Detection of HERV pol homologous sequences suggests enhanced reverse transcriptase activity	Small sample size, limited detection specificity
Duarte et al., 2024 (Duarte et al., 2024)	Brain Tissue	HERV-W, HERV-K	Significant upregulation of HERV-W and HERV-K transcripts	Schizophrenia (Stage not specified)	TWAS (PsychENCODE, CommonMind) found 13 HERV loci linked to schizophrenia risk (P<5×10^-8^), with HERV-W upregulated in glutamatergic neurons.	Uses transcriptomic data, no blood/CSF samples. Unclear patient stage, clinical features; potential medication/other confounders.
Li et al., 2019 (Li et al., 2019)	Brain Tissue (Frontal Cortex, Pons)	HERV-W/H	Transcriptional upregulation		HERV-W/H transcripts upregulated in frontal cortex and pons, associated with disease progression	Regional specificity differences require further study, individual genetic background may influence results
Kim et al., 2008 (Kim et al., 2008)	Brain Tissue (Pons)	HERV-W	Strong env expression		High HERV-W env expression in pons, suggesting involvement in regulation of vital functions	Small sample size, other brain regions not explored
Weis et al., 2007 (Weis et al., 2007)	Brain Tissue (Cingulate Gyrus, Hippocampus)		Reduced GAG protein expression		Reduced HERV-W GAG expression in cingulate gyrus and hippocampus, possibly due to transcriptional defects or antisense transcription	Regional specificity differences need validation, limited sample size

However, viral metagenomics in drug-naive FES patients with predominant negative symptoms revealed no evidence of HERVs involvement (Canuti et al., 2015b). This discrepancy may stem from sample type, cohort size, or the lower neuroinflammatory state in negative-symptom patients, as HERV upregulation is often linked to inflammatory signals or stress responses (Tamouza et al., 2021). Furthermore, inflammation can activate HERVs via epigenetic mechanisms, establishing a positive feedback loop (Perron et al., 2012).

CNS studies reveal HERVs expression in cerebrospinal fluid (CSF) and brain tissue. Retroviral RNA was detected in the CSF and brains of individuals with schizophrenia, with 28.6% of 35 FES patients’ CSF samples showing HERV pol homologous sequences, indicating enhanced reverse transcriptase activity (Karlsson et al., 2001; Yolken, 2004). However, some studies suggest weak correlations between brain HERV pol transcription and schizophrenia, potentially influenced by genetic background, brain-infiltrating immune cells, or medications (Frank et al., 2005). RNA-seq analyses reveal upregulated HERV-W/H transcripts in the frontal cortex and pons of individuals with schizophrenia (Li et al., 2019), supporting a role for HERVs transcriptional activation in disease development. Similarly, HERV-W env is strongly expressed in the pons, a region that regulates vital functions (Kim et al., 2008). Conversely, HERV-W GAG protein expression is reduced in the cingulate gyrus and hippocampus, possibly due to transcriptional defects or antisense transcription (Mack et al., 2004; Mura et al., 2004; Weis et al., 2007). These regional differences underscore the complexity of HERVs expression, which is likely influenced by disease stage and neuroinflammatory status. Recent transcriptome-wide association studies (TWAS) integrating HERVs expression data revealed 13 HERV loci significantly associated with schizophrenia risk (P<5×10^-8^), with HERV-W and HERV-K transcripts upregulated in glutamatergic neurons, linking HERV activity to genetic risk and neurodevelopmental dysregulation (Duarte et al., 2024).

### 4.2 Heterogeneity of HERVs expression across schizophrenia stages

HERVs expression is closely tied to the clinical presentation and disease course of schizophrenia. HERV-K115 insertions are more prevalent in younger-onset patients, suggesting a role in early disease stages (Otowa et al., 2006). FES patients exhibit significantly lower HERV-K methylation levels compared to controls, whereas patients with multiepisode schizophrenia (MES) show normalized methylation. In MES patients, HERV-K methylation correlates positively with chlorpromazine dosage, but not in FES patients, indicating that antipsychotics may modulate HERV methylation (Mak et al., 2019). DNA methylation, a critical epigenetic modification, regulates gene expression by altering chromatin accessibility and is implicated in neurodevelopment and synaptic plasticity (Greenberg and Bourc’his, 2019; Hosak and Hosakova, 2015). Genetic variants associated with DNA methylation are enriched in schizophrenia risk loci during fetal brain development (Hannon et al., 2016). HERV-W env protein positivity is linked to increased manic symptoms and higher chlorpromazine doses, potentially involving inflammatory processes (Queissner et al., 2018; Tamouza et al., 2021). Valproic acid (VPA) upregulates HERV-W and ERV9 transcription in a dose-dependent manner, with HERV-W showing the strongest response in glioblastoma cell lines, while HERV-K (HML-2) transcription remains unaffected (Diem et al., 2012). These findings highlight the complex interplay between HERV expression, disease stage, clinical phenotype, and therapeutic interventions, emphasizing the need to further explore the pathophysiological roles of HERVs in schizophrenia.

## 5 Molecular mechanisms of HERVs in schizophrenia

### 5.1 HERVs activation of neuroinflammatory pathways and programmed cell death

Neuroinflammation has been increasingly recognized as a key contributor to schizophrenia pathogenesis, especially during vulnerable periods of brain development, where prenatal or early-life immune activation can disrupt neural circuits and lead to long-term neurodevelopmental abnormalities (Meyer, 2013). Abnormal activation of HERVs during brain development may represent a potential trigger for inflammatory responses. HERVs may both promote and be activated by inflammation (Helmy and Selvarajoo, 2021; Rangel et al., 2022). Elevated HERV-W env expression in individuals with schizophrenia correlates with proinflammatory cytokines (Tamouza et al., 2021), and downregulation of IL-6 in SH-SY5Y cells inhibits HERV-W env-induced C-reactive protein(CRP) expression (Wang et al., 2018). In mice, prenatal inflammatory exposure induces persistent HERVs expression changes associated with IL-6 (Herrero et al., 2023). Conversely, HERVs exhibit proinflammatory properties; for example, human and rat microglia exposed to HERV-W env show increased proinflammatory cytokine and chemokine production (Kremer et al., 2019; Wang et al., 2021). HERV-W also enhances Th1-like responses via TLR4 activation in monocytes (Rolland et al., 2006). HERV-W env upregulates TNF-α and IL-10 via the TLR4/MyD88 pathway in glial cells, disrupting the proinflammatory/anti-inflammatory balance and contributing to neuroinflammation and synaptic dysfunction (Wang et al., 2021). These findings are consistent with evidence from maternal immune activation models of schizophrenia, where microglial inducible nitric oxide synthase (iNOS) upregulation drives oxidative/nitrosative stress and hippocampal neuronal damage (MacDowell et al., 2017; Ribeiro et al., 2013). HERV-W env further amplifies this by inducing iNOS expression in human microglia-like CHME-5 cells, elevating nitric oxide (NO) production and promoting microglial migration, thereby contributing to neuronal injury (Xiao et al., 2017).

Beyond these direct neurotoxic effects, HERV-W env engages adaptive immune mechanisms, where specific HLA-A*0201-restricted epitopes trigger robust cytotoxic T lymphocyte (CTL) responses, potentially exacerbating neuronal injury through targeted immune attack (Tu et al., 2017). This pathway, distinct from direct cellular effects, involves sustained immune-mediated processes that may perpetuate neuroinflammatory damage over time. Furthermore, HERV-W env engages broader inflammatory cascades, such as cGAS/STING-dependent innate immune activation that promotes neuronal apoptosis (Li et al., 2023). Programmed cell death (PCD), including apoptosis and pyroptosis, is intricately linked to inflammation, where inflammatory signals can trigger PCD pathways as a mechanism to resolve or propagate tissue damage, while dysregulated PCD may in turn amplify inflammatory responses through the release of damage-associated molecular patterns (DAMPs) (Yang et al., 2015). In recent-onset schizophrenia, HERV-W env suppresses linc01930 expression, enhancing cGAS/STING-IRF3 signaling and IFN-β production, which drives innate immune activation and neuronal apoptosis (Li et al., 2023). Similarly, HERV-W env upregulates NLRP3, CASP1, and GSDMD expression, promoting lactate dehydrogenase (LDH) and IL-1β release and inducing CASP1–GSDMD-dependent neuron pyroptosis in recent-onset schizophrenia (Jia et al., 2025). These innate immune pathways intersect with mitochondrial function, where inflammatory signals impair energy metabolism and exacerbate neuronal vulnerability (Buttiker et al., 2022). HERVs amplifies this damage by disrupting mitochondrial function. For instance, ERVWE1, through interaction with CPEB1, downregulates NDUFV2 expression, leading to mitochondrial complex I defects in SH-SY5Y neuroblastoma cells, contributing to neuronal dysfunction in recent-onset schizophrenia (Xia et al., 2021). ERVWE1 upregulates circ_0001810 through AK2 activation, disrupting mitochondrial membrane potential and mitochondrial dynamics, which further compromises neuronal function (Li W. et al., 2024). Additionally, Some researchers suggested that micromitophagy may be involved in schizophrenia pathophysiology, possibly influenced by viral infections that induce mitochondrial autophagy. Specifically, ERVWE1 inhibited micromitophagy by increasing NADPH oxidase activator 1 (NOXA1) expression, which in turn decreases the expression of key micromitophagy-related genes, PTEN-induced kinase 1 (PINK1) and Parkin, and reduces the production of PDHA1-positive TOM20-negative mitochondrial derived vesicles (MDVs) (Zhang et al., 2025). These findings suggest that HERVs-induced inflammation forms a critical link between genetic and environmental risk, with bidirectional feedback loops (Kury et al., 2018). However, some studies report weak associations between systemic inflammation and HERV-W expression, possibly due to nonacute disease stages or limited sample sizes(Sara Coelho Rangel et al., 2024).

### 5.2 HERVs disruption of neurotransmitter systems and synaptic function

HERVs also disrupt neurotransmitter systems and neuronal function. In mice, hippocampal HERV-W env overexpression during development impairs the glutamatergic system, inducing psychosis-related behaviors in adulthood (Johansson et al., 2020). Similarly, HERV-W env enhances dopamine receptor d2 (DRD2) signaling via the protein phosphatase 2A (PP2A)/protein kinase B (AKT1)/glycogen synthase kinase 3(GSK3) pathway, leading to dopaminergic hyperactivity (Yan et al., 2022). HERVs affect neuronal morphology and function; ERVW-1 reduces hippocampal neuron density and impairs dendritic spine morphology in individuals with schizophrenia(W. Yao et al., 2023), contributing to disease pathogenesis. In serotonergic neurons, ERVWE1 reduces neuronal complexity and spine density by upregulating 5-Hydroxytryptamine receptor 1B(HTR1B) (Wu et al., 2023a). Conversely, HERV-W env can activate neurons by reducing 5-HT4Rs, thereby activating small conductance calcium-activated potassium channel 2(SK2) channels, suggesting a novel mechanism for neuronal activity modulation(Wu, Yan, et al., 2023). Collectively, HERVs contribute to a complex neurotoxicity network in schizophrenia by disrupting neurotransmitter balance and impairing neuronal structure.

### 5.3 HERVs’ regulation of epigenetic networks and schizophrenia risk genes

A series of inflammatory responses in the brain may be associated with the aberrant expression of ERVs resulting from the loss of epigenetic co-repressor proteins, such as Trim28 (Jonsson et al., 2021). Numerous psychiatric disorders, including schizophrenia, are recognized as outcomes of neurodevelopmental alterations (Bale et al., 2010; Horwitz et al., 2019). The interplay between genetic predispositions and environmental exposures contributes significantly to the onset and progression of these disorders, highlighting the critical role of epigenetic modifications in disease processes (Khashan et al., 2008). During early development, ERVs are dynamically silenced at the transcriptional level through epigenetic modifications, including histone methylation and deacetylation as well as DNA methylation (Rowe et al., 2010). These repressive mechanisms collectively suppress ERV expression in somatic tissues. Research has indicated that the link between aberrant ERV expression and inflammatory responses in the brain is associated with the loss of Trim28, an epigenetic co-repressor protein. In proliferating neural progenitor cells (NPCs), ERV expression is subject to dynamic regulation dependent on H3K9me3 histone methylation, whereas in cortical neurons of adult mice, Trim28 deficiency leads to elevated ERV expression, accompanied by microglial activation and accumulation of inflammatory proteins (Jonsson et al., 2021). Furthermore, the accumulation of misfolded proteins and the disruption of protein homeostasis can induce endoplasmic reticulum (ER) stress, triggering the unfolded protein response (UPR). ER stress impairs neuroplasticity (Kawada et al., 2014) and is closely associated with metabolic dysregulation in individuals with schizophrenia (Hong et al., 2022; Zhou et al., 2022). Evidence suggests that ERVW-1 downregulates GANAB expression in SH-SY5Y neuroblastoma cells, activating the ATF6-mediated unfolded protein response, which upregulates CHOP and XBP1s, thereby inducing ER stress and impairing protein homeostasis in recent-onset schizophrenia (Xue et al., 2023).

Moreover, HERVs activation influences the expression of schizophrenia risk genes through epigenetic modifications, with evidence suggesting that HERV-mediated transcriptional changes are associated with altered DNA methylation (Chen et al., 2018; Duarte et al., 2024). A human-specific HERV insertion (hsERV_PRODH) serves as an enhancer for the schizophrenia-linked gene *PRODH*, upregulating its expression via low methylation and *SOX2* binding, underscoring the role of HERV in epigenetic and transcriptional regulation (Suntsova et al., 2013). Under physiological conditions, HERVs are silenced by DNA methylation and histone modifications to prevent genomic instability and aberrant immune activation (Geis and Goff, 2020). HERV LTRs serve as sense or antisense promoters (Cohen et al., 2009), regulate host gene expression (Dunn et al., 2006), and drive long noncoding RNAs (e.g., vlincRNAs) that influence pluripotency and tumorigenesis (St Laurent et al., 2013). A full-length HERV-W LTR in the gamma-aminobutyric acid type B receptor 1(GABBR1) regulatory region may induce hypermethylation, downregulating GABBR1 expression (Hegyi, 2013), which is consistent with DNA methyltransferase 1 (DNMT1) overexpression in GABAergic interneurons and reelin promoter hypermethylation in schizophrenia (Grayson et al., 2005; Veldic et al., 2003). Collectively, these methylation-mediated mechanisms highlight the multifaceted role of HERVs in modulating schizophrenia risk genes. Beyond DNA methylation, HERVs also modulate risk gene expression via post-translational phosphorylation pathways, integrating signaling cascades that further dysregulate neuronal function. In U251 glioma cells, HERV-W env overexpression upregulated *BDNF* via glycogen synthase kinase 3 beta(GSK3β) Ser9 phosphorylation (Qin et al., 2016). Similarly, HERV-W env regulates schizophrenia risk genes through phosphorylation-related pathways, activating cAMP response element-binding protein (CREB) phosphorylation to upregulate the expression of the small conductance Ca^2+^-activated K^+^ channel gene (*KCNN2*) in human neuroblastoma cells, thereby modulating neuronal excitability and synaptic signaling (Li et al., 2013). This CREB-dependent mechanism may synergize with GSK3β-mediated pathways, as HERV-W env also enhances CREB phosphorylation to upregulate *BDNF* and dopamine receptor D3 (DRD3), contributing to excitatory-inhibitory imbalances in schizophrenia (Huang et al., 2011). *BDNF*, a neurotrophin critical for neuronal survival, migration, differentiation, and synaptic plasticity (Guo et al., 2010), is regulated by *DISC1*, a schizophrenia risk gene modulated by HERV-W env through calcium-dependent Transient receptor potential canonical 3(TRPC3) channel activation (Chen et al., 2018). The *DISC1-GSK3β-BDNF* axis may mediate the pathological effects of HERVs.

In summary, the molecular mechanisms underlying HERVs involvement in schizophrenia reveal a multifaceted interplay of immune-mediated neuroinflammation, programmed cell death, neurotransmitter dysregulation, and epigenetic modulation of risk genes, collectively bridging genetic vulnerabilities with environmental triggers to perpetuate synaptic dysfunction and neurodevelopmental deficits. These insights not only underscore HERVs’ potential as biomarkers, but also highlight opportunities for targeted therapies.

## 6 HERVs as mediators of schizophrenia risk factors

The risk factors for schizophrenia include genetic predispositions, infections, and social stressors (Davis et al., 2016). The ‘viral hypothesis’ posits that prenatal/perinatal or postnatal viral infections, or immune responses to them, impair brain maturation, leading to psychotic symptoms in adolescence (Canuti et al., 2015a). Supporting evidence includes elevated maternal IL-8 levels linked to the risk of schizophrenia in offspring (Brown et al., 2004) and a 5–8% increased risk for individuals born in winter/spring, when infections are prevalent (O’Callaghan et al., 1991). Persistent or reactivated dormant viral infections during adolescence may also contribute (Kotsiri et al., 2023). HERVs, as retroviruses, may directly contribute to schizophrenia or be activated by other viruses, such as influenza or herpes simplex virus type 1, which upregulate HERV-W env transcription (Nellaker et al., 2006; Ruprecht et al., 2006). Influenza infection activates ERVWE1 by increasing Glial cells missing homolog 1(*GCM1*) transcription and reducing repressive histone marks (H3K9me3)(F. Li et al., 2014), whereas SARS-CoV-2 upregulates HERV-W env in lymphoid cells (Charvet et al., 2023), highlighting the role of HERVs as a bridge in virus-mediated schizophrenia pathogenesis.

Environmental stressors also stimulate HERVs expression. HERV-W env antigenemia is significantly more common in individuals with schizophrenia and correlated with childhood trauma, suggesting that early adversity is a trigger for HERV reactivation (Tamouza et al., 2021). Pharmacological agents, such as caffeine and aspirin, increase HERV-W env and gag expression in SH-SY5Y neuroblastoma cells (Liu et al., 2013). However, some studies argue that HERVs activation directly contributes to disease causation, not merely as a compensatory or environmentally triggered response. A TWAS of the dorsolateral prefrontal cortex identified 163 significant risk expression traits in schizophrenia, with 15 (9%) HERVs-related traits, including 9 upregulated and 6 downregulated features associated with genetic risk (Duarte et al., 2024). Thus, HERVs may directly contribute to schizophrenia risk or act as a bridge between genetic and environmental factors, emphasizing their critical role in the complex etiology of this disease.

## 7 Discussion

HERVs are emerging as key players in schizophrenia, with clinical evidence, molecular mechanisms, and risk factor associations highlighting their importance. Mounting evidence from numerous studies has demonstrated aberrant HERVs expression, particularly of HERV-W and HERV-K expression, in the peripheral blood, cerebrospinal fluid, and brain tissues of patients with schizophrenia, with elevated env and gag transcripts frequently observed in these patients compared with healthy controls. These findings position HERVs as potential biomarkers for schizophrenia diagnosis and prognosis, particularly in first-episode and acute-phase patients. Moreover, HERVs activation interacts with environmental factors, such as viral infections, childhood trauma, and pharmacological interventions, underscoring their role as a nexus between genetic and environmental risk. HERVs contribute to schizophrenia pathogenesis through neuroinflammatory pathways, neurotoxicity, and the dysregulation of risk genes (e.g., *BDNF*, *DISC1*, *PRODH*) via epigenetic and transcriptional mechanisms. Notably, HERV-W env-mediated activation of the TLR4/MyD88 pathways and its impact on glutamatergic and dopaminergic signaling highlight their multifaceted role in neuroinflammation, synaptic dysfunction, and neuronal apoptosis.

Despite these advances, challenges persist in elucidating the precise roles of HERVs. Inconsistent findings, potentially attributable to variations in sample types (e.g., blood vs. CSF), disease stages (e.g., FES vs. MES), and methodological differences, underscore the need for standardized HERV-specific assays. Whether HERVs activation is a cause or consequence of schizophrenia remains unresolved, with evidence suggesting bidirectional feedback loops involving inflammation and epigenetic dysregulation. Small sample sizes and patient heterogeneity limit statistical power, necessitating larger, longitudinal studies to track HERVs expression across disease stages and correlate it with clinical phenotypes and biomarkers (e.g., cytokines and neurotransmitter metabolites).

Future research should leverage advanced sequencing technologies, such as long-read and single-cell RNA sequencing, to map HERVs expression at specific genomic loci and cell types, potentially identifying novel therapeutic targets. Preclinical studies targeting HERV-W env, inspired by monoclonal antibodies such as temelimab in multiple sclerosis, could inform similar interventions in schizophrenia. Correlating HERVs expression with epigenetic markers (e.g., DNA methylation and histone modifications) may elucidate regulatory mechanisms and facilitate the development of biomarker panels for early diagnosis. Additionally, investigating the effects of exploring environmental triggers (e.g., infections and stress) on HERVs activation could clarify gene-environment interactions, guiding preventive strategies.

In conclusion, HERVs represent a critical intersection of genetic, epigenetic, and environmental factors in schizophrenia, offering a unique lens through which to investigate its complex etiology. Addressing methodological inconsistencies, expanding cohort studies, and leveraging cutting-edge genomic tools will be essential to unravel the pathomechanisms of HERVs and translate these insights into actionable targets for innovative diagnostics and therapies in schizophrenia management.
